# The Clinical Challenges of Diagnosing Acutely Decompensating Amyloidosis

**DOI:** 10.7759/cureus.11418

**Published:** 2020-11-10

**Authors:** Karim Bayanzay, Alejandra Razzeto, Behzad Amoozgar, Pavan Garala, Alissa Holman

**Affiliations:** 1 Internal Medicine, Jersey Shore University Medical Center, Perth Amboy, USA

**Keywords:** amyloidosis, cardiac amyloidosis, al amyloidosis, congo red, biopsy, nephrotic syndrome

## Abstract

Advanced amyloidosis and related multi-organ manifestations are devastating clinical scenarios. Because ambiguous presentation of amyloidosis may occur, early diagnosis and prevention of organ damage, such as cardiac injury, is essential and requires high clinical intuition. Our patient was a middle-aged female with a past medical history of heart failure with several decompensation episodes who presented with jaundice, itchiness, and weight loss. Further workup revealed pulmonary hypertension, restrictive heart disease, possible underlying obstructive liver disorder, and hyperkalemia. During admission, the patient established bradycardia and required a pacemaker temporarily, and later she manifested atrial fibrillation. Liver biopsy primarily was suggestive of hepatic congestion. Unfortunately, the patient died during workup due to cardiac arrest. Premortem laboratory results were suggestive of amyloidosis, which was confirmed later by re-examining the liver biopsy with Congo red. Diagnosis of amyloidosis requires early clinical suspicion and workup to prevent its progression to fatal organ involvement such as cardiac complications.

## Introduction

The diagnosis of amyloidosis is very challenging, as its presentation can be vague and highly variable. Incidental diagnosis of amyloidosis is also rare since there are no pathognomonic findings on routine imaging or serum tests [1,2]. Therefore, clinical suspicion for amyloidosis must remain elevated, and appropriate testing must be performed in a timely fashion to diagnose the condition. Here, we will present an unfortunate case of a woman with an uncommon presentation of amyloidosis that was not discovered until it was too late.

## Case presentation

The patient was a 55-year-old female presenting to the emergency room complaining of itching and jaundice for the past one month, which was nonresponsive to diphenhydramine. The patient reports approximately 15-pound weight loss over the last month, which she attributes to furosemide. The patient denied any change in appetite, abdominal pain, abdominal distention, fever, or altered mental status. There was no history of alcohol abuse, renal disease, or liver disease, but a remote history of cholecystectomy.

Over the past eight months, the patient had multiple admissions for heart failure decompensation. The previous echocardiogram showed reduced ejection fraction (40-45%), left ventricular hypertrophy with abnormal diastolic dysfunction, and biatrial dilation. Transmitral Doppler flow was consistent with a restrictive pattern suggestive of high left atrial pressure. Pulmonary hypertension was also noted with elevated right ventricular systolic pressure (50-60 mmHg). A pharmacological stress test at the time did not show ischemic changes. The patient’s heart failure was being managed with furosemide 40 mg twice daily, lisinopril 10 mg daily, spironolactone 25 mg daily, and metoprolol tartrate 50 mg twice daily.

Upon presentation to our facility, the patient's initial vitals were as follows: blood pressure was 133/54, heart rate was 67 beats/minute, temperature was 97.4 Fahrenheit, and oxygen saturation was 97% on room air.

On physical examination, the patient had severe jaundice with scleral icterus and prominent scratch marks across arms, legs, and abdomen. The liver was palpable, but no abdominal distention or tenderness was present, and bowel sounds were normal. Murphy’s sign was negative. Auscultation revealed a healthy heart and breath sounds, and no raised jugular venous pressure and no peripheral edema were noted.

**Figure 1:**
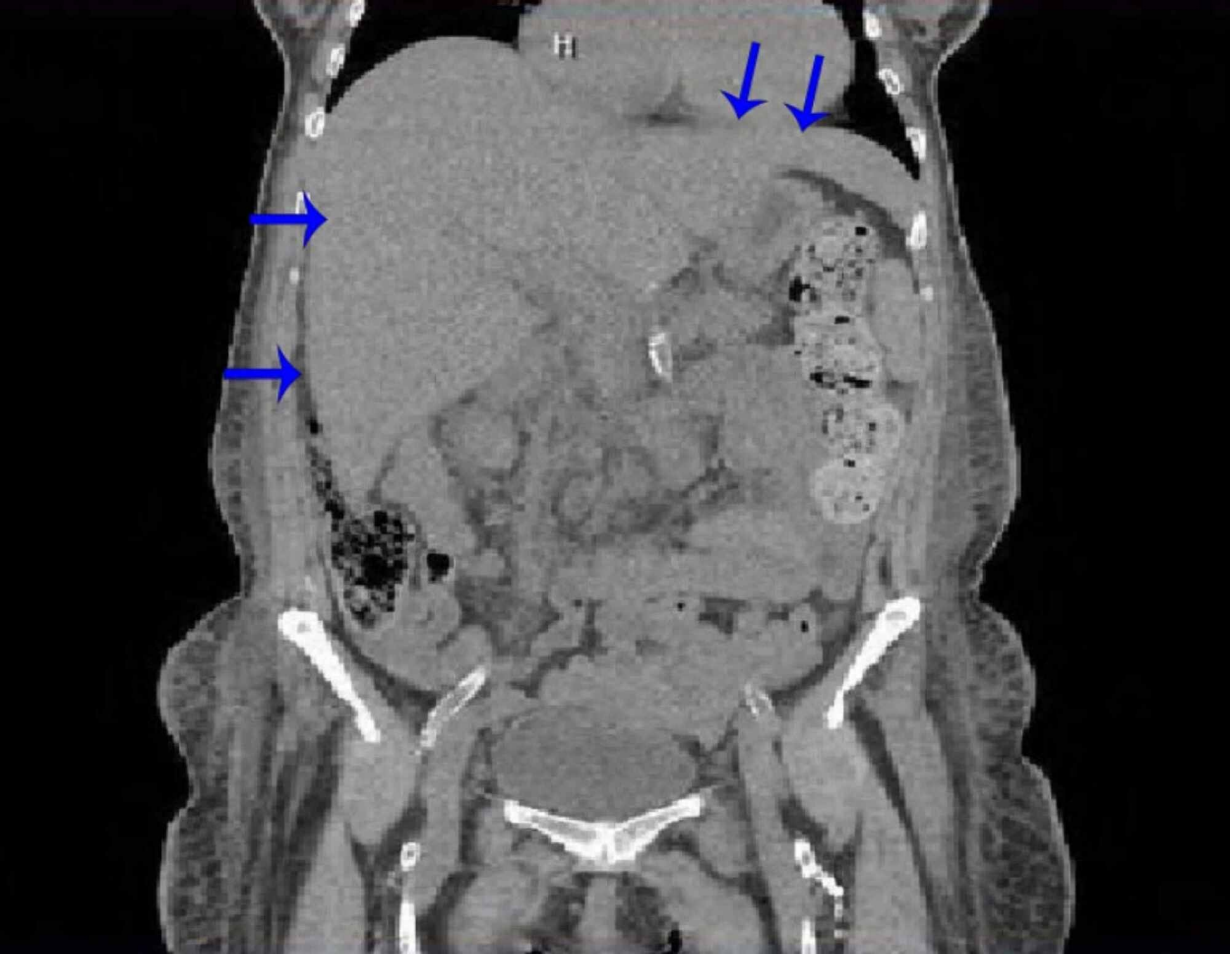
CT of the abdomen showing hepatomegaly

**Figure 2:**
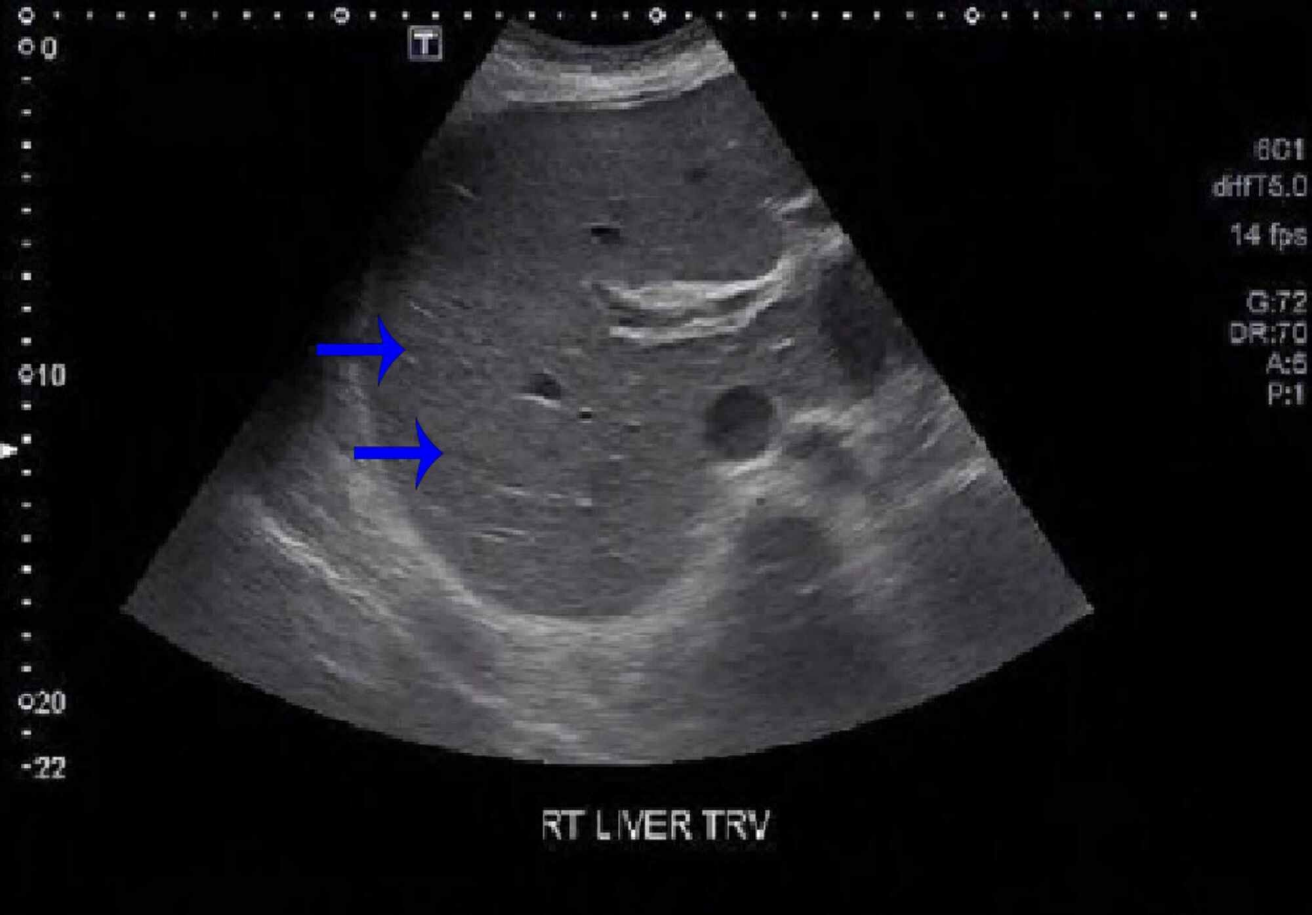
Abdominal ultrasound showing hepatic parenchymal disease

As shown in Table 1, laboratory studies showed findings consistent with obstructive jaundice (total bilirubin: 17.3 mg/dL; direct bilirubin: 13.6 mg/dL; alkaline phosphatase: 924 U/L; gamma glutamyl-transferase: 368 U/L) with mild elevation in liver enzymes (AST [aspartate aminotransferase]: 78 U/L; ALT [alanine aminotransferase]: 59 U/L). The viral and autoimmune hepatitis panel was negative. Electrolytes showed hyponatremia (130 mmol/L), hyperkalemia (5.8 mmol/L), and mildly elevated creatinine (1.4 mg/dL). Urinalysis was normal upon admission. Computed tomography (CT) of the abdomen on admission showed hepatomegaly without biliary dilation and minimal ascites (Figure 1). Abdominal ultrasound showed hepatic parenchymal disease with no biliary ductal dilation and unremarkable kidneys (Figure 2).

**Table 1:** Laboratory Results on Admission INR, international normalized ratio; WBC, white blood count; MCV, mean corpuscular volume; BUN, blood urea nitrogen; GGT, gamma glutamyl-transferase; AST, aspartate aminotransferase; ALT, alanine aminotransferase; Ag, antigen; Ab, antibody

Laboratory workup	Values	Reference
INR	1.1	0.9–1.1
WBC (x10^3^/mL)	13.9	3.6–11
Hemoglobin (g/dL)	9.2	10.3–15.1
MCV (fL)	79.9	73.5–96.5
Platelets (x10^3^/uL)	137	150–372
BUN (mg/dL)	36	6–20
Creatinine (mg/dL)	1.4	0.5–0.9
Sodium (mmol/L)	130	135–145
Potassium (mmol/L)	5.8	3.5–5.0
Alkaline phosphatase (U/L)	924	40–130
GGT (U/L)	368	10–40
Albumin (g/dL)	2.9	3.5–5.0
Total bilirubin (mg/dL)	17.3	0.0–2.0
Direct bilirubin (mg/dL)	13.6	0.1–0.3
AST (U/L)	78	15–40
ALT (U/L)	59	5–30
Haptoglobin (mg/dL)	111	30–200
Hepatitis A Ab	Negative	
Hepatitis B surface Ab	Negative	
Hepatitis B surface Ag	Negative	
Hepatitis B core Ab	Negative	
Hepatitis C Ab	Negative	

**Figure 3:**
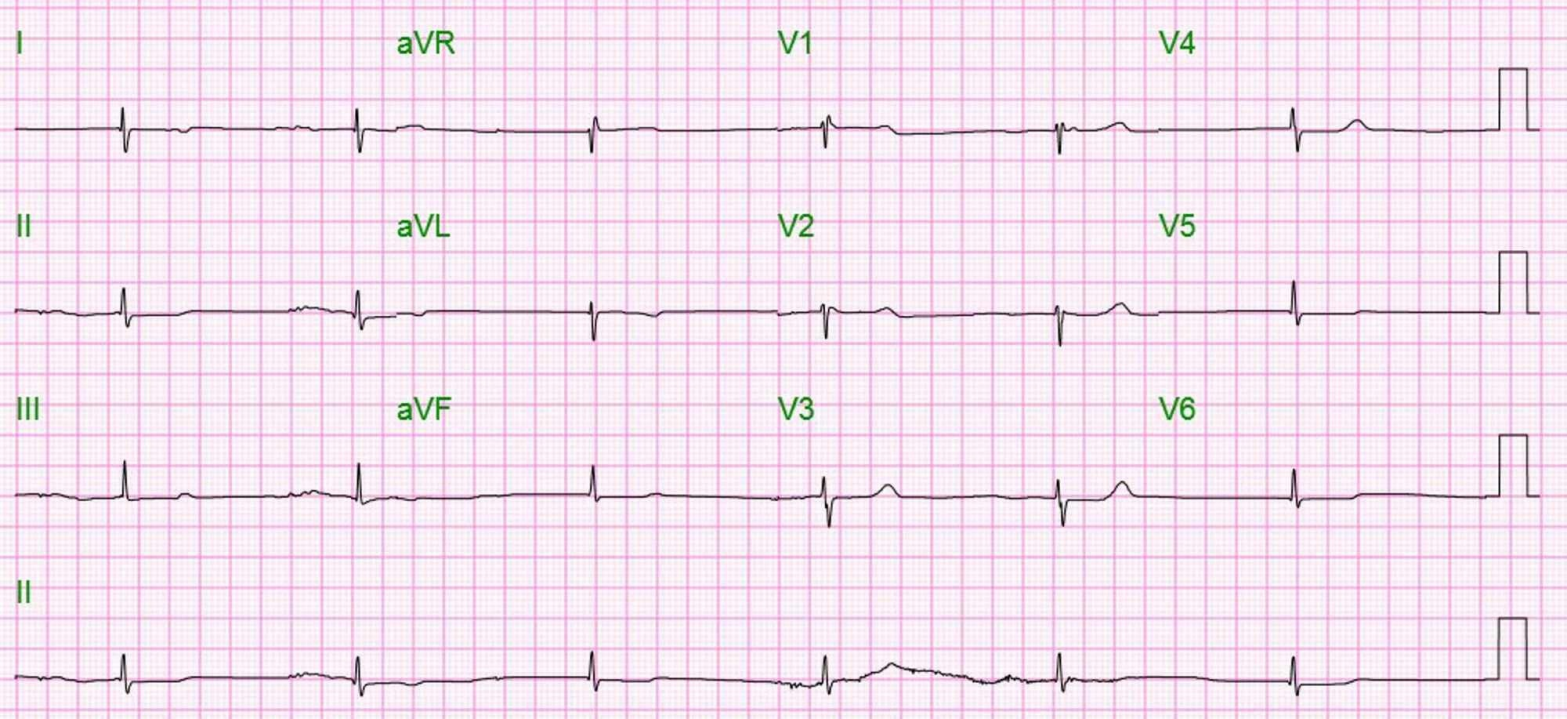
EKG showing bradycardia secondary to junctional rhythm EKG, electrocardiogram

During her admission, her bilirubin continued to increase steadily. After two days, she had an episode of symptomatic bradycardia requiring transcutaneous pacing with an electrocardiogram (EKG) that showed a junctional escape rhythm (Figure 3).

**Figure 4:**
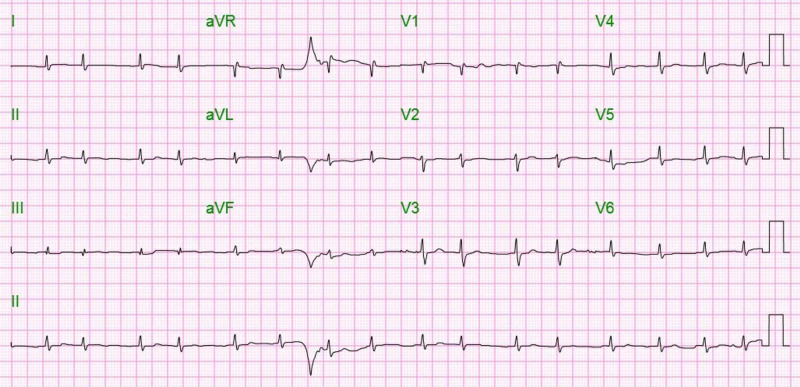
EKG showing atrial fibrillation and low voltage limb leads EKG, electrocardiogram

At the same time, the patient’s hyperkalemia continued to worsen, and therefore it was assumed that bradycardia was secondary to a combination of hyperbilirubinemia and hyperkalemia. The patient’s creatinine steadily increased and hyperkalemia was not responding to treatment; therefore, a nephrologist started dialysis. At the time, her worsening renal function was attributed to hepatorenal syndrome, and further renal workup was not performed. In the meantime, the patient was started on a transcutaneous pacemaker, which was removed a few days later, when the patient developed atrial fibrillation (Figure 4).

The liver biopsy showed marked dilatation of hepatic veins and hepatic sinusoids with bile stasis in hepatocytes and intrahepatic biliary ducts, consistent with hepatic congestion. Since the liver parenchyma was healthy, this pattern suggested that her liver disease was secondary to cardiac dysfunction rather than an intrinsic liver pathology. Following this liver biopsy report, the diagnosis of hepatorenal syndrome to explain her renal disease was questioned. The nephrologist recommended a kidney biopsy and laboratory workup for nephrotic syndrome, as he noted nephrotic range proteinuria. Unfortunately, the patient passed away unexpectedly before the kidney biopsy could be performed. The immediate cause of death appears to be cardiogenic shock secondary to fluid overload; however, the family refused autopsy to confirm.

Before passing away, laboratory workup to evaluate for nephrotic syndrome was collected and sent. Anti-nuclear antibodies (ANA) and anti-histone antibodies were positive. Myeloperoxidase-antineutrophil cytoplasmic antibodies (MPO-ANCA) titer as well as elevated perinuclear anti-neutrophil cytoplasmic antibodies (p-ANCA) titer were elevated (Table 2).

**Table 2:** Serum Markers ANA, anti-nuclear antibodies; PLA2R, phospholipase A2 receptor; MPO-ANCA, myeloperoxidase-antineutrophil cytoplasmic antibodies; p-ANCA, perinuclear anti-neutrophil cytoplasmic antibodies; IgA, immunoglobulin A; GBM, glomerular basement membrane; IgG, immunoglobulin G

Laboratory workup	Results	Reference
ANA titer	1:2560	<1:80
Anti-histone (units)	1.7	0.0–0.9
Anti-PLA2R antibody	<1:10	<1:10
MPO-ANCA (AU/mL)	30.0	0–19
Serine protease 3 (AU/mL)	4.0	0–19
p-ANCA	1:320	<1:20
Anti-smooth muscle antibody	1:20	<1:20
Anti-IgA (U/mL)	<99	<99
Complement 4 (mg/dL)	20	10–40
Complement 3 (mg/dL)	87	88–201
Kappa free (mg/dL)	18.40	0.33–1.94
Lambda free (mg/dL)	16.20	0.57–2.63
Kappa/lambda free ratio	1.14	0.26–1.65
Anti-mitochondrial antibodies (units)	6.9	0–20
Anti-GBM IgG (AU/mL)	0	0–19

**Figure 5:**
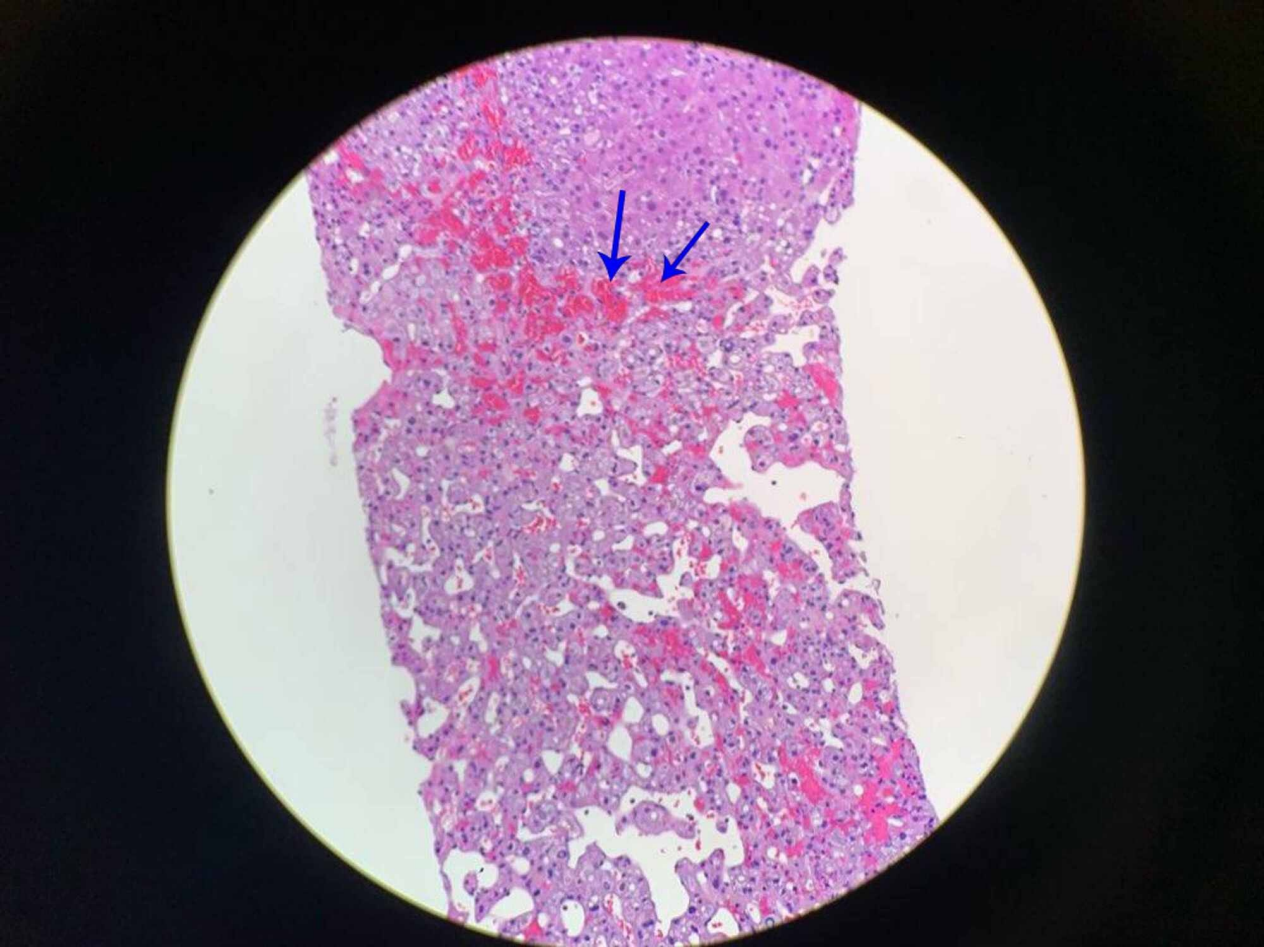
Liver biopsy with Congo red stain

Kappa/lambda was elevated, while serum protein electrophoresis and immunofixation (Table 3) showed significantly elevated IgM. Afterward, the liver biopsy was further re-examined with Congo red stain, and amyloidosis was confirmed (Figure 5).

**Table 3:** Serum Protein Electrophoresis Ig, immunoglobulin

Laboratory workup	Results	Reference
Total protein (g/dL)	7.40	6.00–8.30
Albumin (g/dL)	3.54	3.75–5.01
Alpha 1 globulin (g/dL)	0.53	0.19–0.46
Alpha 2 globulin (g/dL)	0.70	0.48–1.05
Beta globulin (g/dL)	1.74	0.62–1.51
IgG (mg/dL)	1280	768–1632
IgA (mg/dL)	396	68–408
IgM (mg/dL)	374	35–263

## Discussion

Diagnosis and early management of amyloidosis are vital. Unfortunately, an unusual presentation and anchoring bias prevented the elucidation of the underlying condition early enough to prevent death.

The enigmatic presentation of obstructive jaundice was the first red herring that led the clinicians astray. Based on initial examination and laboratory findings, we assumed it to be of a primary intrahepatic source. Therefore, initial investigations were directed towards identifying the cause of intrinsic liver disease. Primary biliary cirrhosis was considered, but the lack of anti-mitochondrial antibodies made such a diagnosis unlikely. Since ANAs and anti-histone antibodies were positive, drug-induced systemic lupus erythematosus (SLE) was also contemplated; but the lack of substantial elevation in liver enzymes made such a scenario improbable. Also, jaundice associated with SLE is more commonly due to hemolysis, and the bilirubin distribution was more in favor of an obstructive etiology rather than a pre-hepatic etiology. Since biomarkers for common causes of intrinsic liver disease were non-revelatory, we pursued a liver biopsy.

Around the same time, there was a development of inexplicable renal disease with treatment-resistant electrolyte disturbances requiring dialysis. There was no history of renal disease, but rather than re-evaluating our assumptions and searching for other causes of renal disease, we assumed it was secondary to hepatorenal syndrome. This assumption was maintained until the liver biopsy uncovered evidence of liver congestion, most likely secondary to restrictive heart disease.

With the updated information, we turned our attention to a search for a primary renal cause and discovered that the patient had nephrotic range proteinuria, a common finding in amyloidosis [3]. At this time, we tested the serum for the presence of immunoglobulin (Ig) light chains and other serum antibodies associated with nephrotic syndrome. Laboratory findings showed positive MPO-NCA), which is typically associated with amyloid A (AA) amyloidosis [4], rather than amyloid light-chain (AL) amyloidosis. Therefore, it is unlikely that the MPO-ANCA contributed to the development of this patient’s amyloidosis. We also found an elevated IgM monoclonal gammopathy, which is more commonly associated with AL amyloidosis [5]. A renal biopsy was not performed, but rapidly progressive glomerulonephritis secondary to amyloidosis is consistent with the clinical course.

Even after a liver biopsy, we failed to diagnose amyloidosis due to lack of an appropriate stain, Congo red, which further highlights the difficulty in diagnosing amyloidosis. To correctly diagnose, not only do we need a biopsy, but suspicion should be also sufficiently elevated to make a special request of Congo red staining. Unfortunately, this was not done until after the patient had expired, and therefore the initial liver biopsy failed to reveal amyloidosis. The amyloid tissue in a biopsy using Congo red staining and the presence of kappa or lambda producing cells established the final diagnosis of AL amyloidosis in this patient [1,6].

In hindsight, there were several characteristics findings of cardiac amyloidosis, which, if interpreted prudently, could have prompted earlier evaluation for amyloidosis. The echocardiogram findings were consistent with amyloidosis-related cardiac involvement such as left ventricular hypertrophy with biatrial enlargement. The low voltage limb leads on EKG (Figure 2) and conduction abnormalities were also suggestive of cardiac amyloidosis [7]. Up to 50% of patients with AL amyloidosis have evidence of cardiac involvement. Cardiac involvement is a particularly ominous sign, with average prognosis ranging between six and eight months [8].

When cardiac amyloidosis remains undiagnosed, it is typically managed with standard heart failure medications, often including beta-blockers, angiotensin-converting enzyme inhibitors (ACE)/angiotensin II receptor blockers (ARB), or calcium channel blockers. Although beneficial to heart failure patients, they can be of limited benefit, or even detrimental, if the heart failure is secondary to cardiac amyloidosis. Beta-blockers are not well tolerated in such patients, as they are prone to conduction abnormalities and their cardiac output becomes heart rate dependent [9]. Calcium channel blockers have been shown to concentrate in amyloidosis tissue, which increases the risk of toxicity [10]. ACE Inhibitors have questionable benefits and may induce severe hypotension.

Since the treatments are contradictory, it is essential to rule out cardiac amyloidosis in the presence of restrictive heart disease, especially if not tolerating or responding to typical heart failure therapy [11]. If diagnosed early enough, chemotherapeutic regimens such as cyclophosphamide, bortezomib, and dexamethasone may have been beneficial. Therefore, without understanding the underlying etiology of heart failure, cardiac amyloidosis is often mismanaged [12,13].

## Conclusions

This was a case report of a patient who died from an undiagnosed AL cardiac Amyloidosis with an underlying IgM monoclonal gammopathy. Her amyloidosis caused a restrictive heart disease, leading to obstructive jaundice and itching. Subsequent renal disease and cardiac conduction abnormalities were incorrectly attributed to liver disease. Workup for amyloidosis was not performed until nephrotic range proteinuria was discovered. In hindsight, echocardiographic and EKG findings were suggestive of amyloidosis but were not aptly interpreted due to lack of suspicion. Although extensive workup had been performed, lack of clinical suspicion and anchoring delayed the diagnosis until after her death. Therefore, clues for amyloidosis should be detected early, and appropriate tests for amyloidosis should be performed to provide treatment in a timely manner.
